# Multiregion single‐cell sequencing reveals the transcriptional landscape of the immune microenvironment of colorectal cancer

**DOI:** 10.1002/ctm2.253

**Published:** 2021-01-01

**Authors:** Wei Wang, Yu Zhong, Zhenkun Zhuang, Jiarui Xie, Yueer Lu, Chengzhi Huang, Yan Sun, Liang Wu, Jianhua Yin, Hang Yu, Zhiqiang Jiang, Shanshan Wang, Chunqing Wang, Yuanhang Zhang, Yilin Huang, Chongyin Han, Zhenggang Zhong, Jialin Hu, Ying Ouyang, Huisheng Liu, Mengya Yu, Xiaochan Wei, Dandan Chen, Lizhen Huang, Yong Hou, Zhanglin Lin, Shiping Liu, Fei Ling, Xueqing Yao

**Affiliations:** ^1^ School of Biology and Biological Engineering South China University of Technology Guangzhou Guangdong China; ^2^ Department of General Surgery, Guangdong Provincial People's Hospital, Guangdong Academy of Medical Sciences, School of Medicine, South China University of Technology Guangzhou Guangdong China; ^3^ BGI‐Shenzhen Shenzhen China; ^4^ China National GeneBank BGI‐Shenzhen Shenzhen China; ^5^ Shenzhen Key Laboratory of Single‐Cell Omics Shenzhen China; ^6^ The Guangdong‐Hong Kong Joint Laboratory On Immunological And Genetic Kidney Diseases Guangdong Provincial People's Hospital, Guangdong Academy of Medical Sciences Guangzhou Guangdong China

## Abstract

The tumor microenvironment is a complex ecosystem formed by distinct and interacting cell populations, and its composition is related to cancer prognosis and response to clinical treatment. In this study, we have taken the advantage of two single‐cell RNA sequencing technologies (Smart‐seq2 and DNBelab C4) to generate an atlas of 15,115 immune and nonimmune cells from primary tumors and hepatic metastases of 18 colorectal cancer (CRC) patients. We observed extensive changes in the proportions and functional states of T cells and B cells in tumor tissues, compared to those of paired non‐tumor tissues. Importantly, we found that B cells from early CRC tumor were identified to be pre‐B like expressing tumor suppressors, whereas B cells from advanced CRC tumors tended to be developed into plasma cells. We also identified the association of IgA^+^IGLC2^+^ plasma cells with poor CRC prognosis, and demonstrated a significant interaction between B‐cell and myeloid‐cell signaling, and found *CCL8* ^+^ cycling B cells/*CCR5^+^* T‐cell interactions as a potential antitumoral mechanism in advanced CRC tumors. Our results provide deeper insights into the immune infiltration within CRC, and a new perspective for the future research in immunotherapies for CRC.

## Graphical abstract

Integrated single‐cell RNA sequencing technologies reveal a high‐resolution immune landscape of colorectal primary tumors and liver metastasis, identifying major immune cell types and distinct cell functional states of T and B cells as well as predictions of complex cell‐cell interactions.

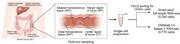

## 1 INTRODUCTION

The tumor microenvironment (TME) consists of a cell component made up of various cell types, including immune cells, inflammatory cells, adipocytes, fibroblasts, and vascular endothelial cells, and noncellular components in and around the tumor.^1^ The cellular components within the TME are emerging as key regulators of primary tumor progression, organ‐specific metastasis, and therapeutic response.^2^ Tumor infiltrating immune cells are the key components,^1^, ^3^ including T lymphocytes, B lymphocytes, myeloid cells, mast cells, and natural killer (NK) cells, among others, which form an ecosystem that modulates all aspects of tumor development.^3^ Among these, T cells are the most abundant and best‐characterized immune cells in the TME of solid tumors.^1^, ^3^ The presence of *CD4* ^+^ T helper 1 (Th1) and cytotoxic *CD8* ^+^ T lymphocytes can prevent tumor growth by targeting antigenic tumor cells, and high densities of activated *CD8* ^+^ T cells within the tumor niche are associated with favorable prognoses in various cancers.^3^ Deeper understanding of the immune complexity of TME will help to identify advanced biomarkers, and allow for devising novel immunotherapy strategies.^4^ Along this line, the single‐cell technology has rapidly become a powerful approach for the analysis of TME, including that of colorectal adenocarcinoma (CRC).^5^

CRC is the fourth leading cause of cancer‐related mortality worldwide, and the prognosis for CRC patients who experience recurrence or metastasis is extremely poor.^6^, ^7^ Surgery, radiotherapy, and chemotherapy have long been the leading strategies for CRC patients. However, the therapeutic effect has never been satisfying, especially for advanced patients with metastatic lesions. In the past decade, immunotherapy has emerged as a potentially effective systemic treatment for advanced CRC patients. So far a great focus has been placed on tumor‐infiltrating T lymphocytes (TILs) as they can directly affect prognosis and the response to immunotherapy.^8^ Several studies have demonstrated that the type, location, and density of TILs can be used to predict the overall survival (OS) and progression‐free survival (PFS) of CRC patients via an immune score strategy.^9^, ^10^ Using single‐cell technology, Zhang et al.^8^ first analyzed the T‐cell subpopulations, and illustrated the distinct TIL cell landscape of CRC. Li et al.^11^ and De Vries et al.^12^ characterized the immune cells and depicted the immune landscape of primary colorectal tumors and matched normal mucosa, respectively. More recently, Zhang et al.^5^ used two single‐cell sequencing methods to characterize the immune and stromal populations of CRC primary tumors in human. However, most of these studies on the TME of CRC have largely focused on T cells or myeloid cells, whereas a comprehensive atlas of tumor‐infiltrating B cells is largely lacking.^8^, ^12^, ^13^ Since the immune system of the gastrointestinal mucosa is chronically challenged by dietary antigens and bacteria, and immune protection driven by antigens in the gastrointestinal mucosa is mainly attributed to antibody‐producing B cells and plasma cells,^14^, ^15^ the characterization of B cells in CRC should provide new insights into the immune ecosystem of CRC. Additionally, CRC metastasizes to the liver in over half of patients,^16^ compared to that of primary tumors, but the biology of CRC liver metastasis (CLM) is currently poorly characterized.

To better understand the immune ecosystem of CRC primary tumor, and with an emphasis on B cells, we performed full‐length scRNA‐sEquation (Smart‐seq2) on ∼6,000 immune cells from primary tumor (included the center area [CT] and the invasive margin area [MT] of tumor), adjacent normal mucosa tissues (ANT), distinct normal mucosa tissues, hepatic metastatic tumors (Cm), and paired noncancerous tissue (Pm) of eight CRC patients. Furthermore, to gain a comprehensive understanding of the immune cellular landscape of hepatic metastases, we also performed full‐length scRNA‐sEquation (Smart‐seq2) on ∼400 cells from paired Cm and Pm. To verify the full‐length scRNA‐seq results, we utilized a high‐throughput platform recently developed in‐house, DNBelab C4, which is a scalable and cost‐effective approach for microfluidic droplet‐based 3′ scRNA‐seq technology^17^ to obtain high‐quality data for 10,000 cells from various tissues in 10 CRC patients. In this study, we found that the discrepancies in previous studies of B cells in tumor may be due to the heterogeneity of B‐cell subtypes in cancer and noncancerous tissues. We demonstrated that IgA^+^IGCL2^+^ plasma cells were associated with poor prognosis in CRC. Paired ligand‐receptor analyses demonstrate that myeloid cells and certain B‐cell subsets could regulate multiple T‐cell subsets in CRC tumor via various ligand‐receptor pairs. This study provides deeper insights into the immune infiltration within CRC.

## 2 RESULTS

### 2.1 A single‐cell transcription atlas of CRC primary tumors and hepatic metastases

**FIGURE 1:**
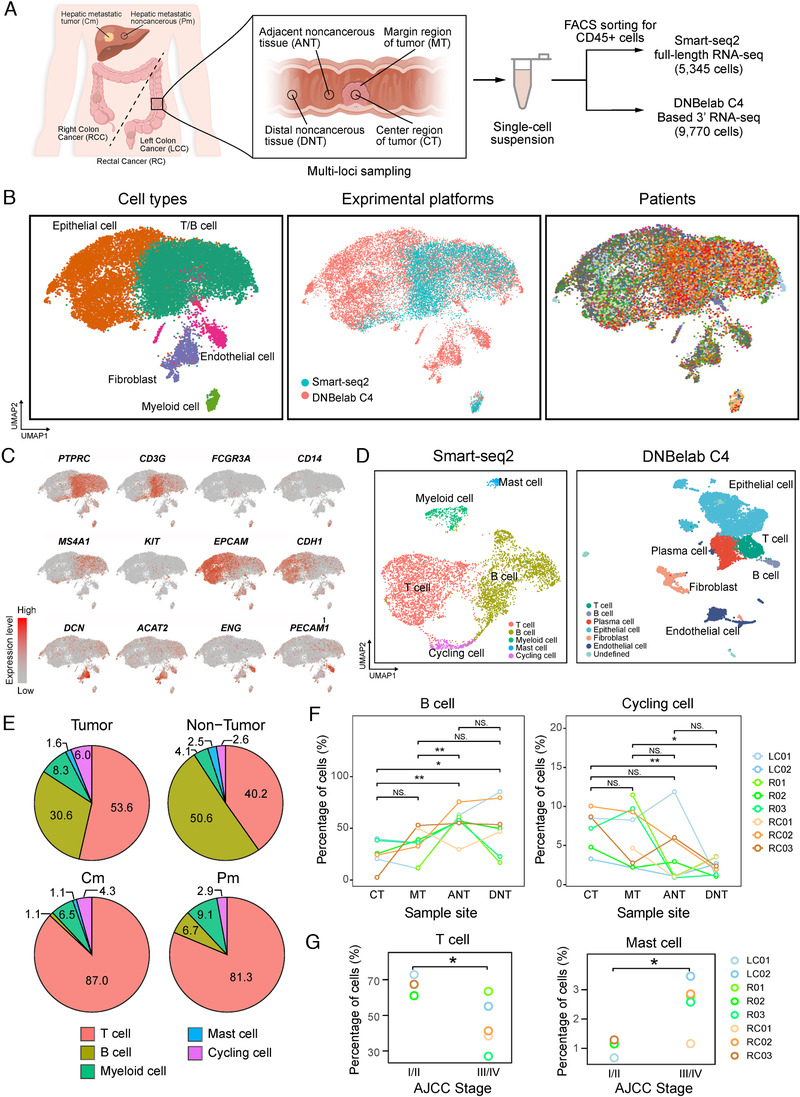
Overview of the immune landscape of colorectal adenocarcinoma (CRC) tumors and nontumor samples. A, Overview of the study design. Colon or rectum tumors and noncancerous samples were collected from 18 patients with CRC. B, Uniform Manifold Approximation and Projection algorithm (UMAP) plot of all cells form two experiment platforms profiled here. Each cell is color‐coded by (left to right): associated cell type, experiment platforms, and patient. C, UMAP plot, color‐coded for the expression level (gray to red) of marker genes in each cell type. D, UMAP plots show clustering results of different experimental platforms. E, Cell type distribution of tumor and nontumor tissues as well as paired hepatic metastatic tumor and nontumor tissues in discovery cohort. F, The percentage of B cells and cycling cells in different regions. G, Comparison of the cell distribution of T cells and mast cells and AJCC stage. NS, *P* > 0.05; *P < 0.05; **P < 0.01; ***P < 0.001, as determined by Student's *t*‐test.

Combining two single‐cell RNA sequencing technologies (Smart‐seq2 and DNBelab C4), we investigated different cell populations within primary tumors of 18 CRC patients and within matching hepatic metastasis from three of these patients (Figure 1A, Table S1, see Section 4). Specifically, for patients RC01, RC02, RC03, R01, R02, R03, LC01, and LC02, a modified full‐length Smart‐seq2 method was used to study the composition of CD45^+^ cells isolated from tumors, ANT (proximal to the brim of carcinoma 3‐5 cm), and distinct normal tissues (DNT, distal to the brim of carcinoma >10 cm). Besides, paired Cm and adjacent liver (Pm) from patient LC02 were also included. For patients NRC01, NR01, NLC03, NLC04, NLC05, NLC06, NLC07, NLC08, NLC09, and NLC10, a recently developed method based on microfluidic droplet method (DNBelab C4)^17^ was used to analyze the cells isolated from tumors, ANT, DNT, Cm, and Pm. After quality control and removing low‐quality cells (see Section 4), 5,345 CD45^+^ single cells (Smart‐seq2) and 9,770 single cells (DNBelab C4) were retained for subsequent analyses. The clinical information and the numbering of cells from each patient are provided in Table S1.

To define the major cell populations within CRC primary and hepatic metastases, we first performed graph‐based cell clustering^19^ on these two datasets, after removing batch effects among multiple samples (see Section 4). Based on the expression of canonical cell lineage markers (Figure 1C, Figure S1A and S1B), we identified seven major cell types, including four immune cell types (T cells, B cells, myeloid cells, and mast cells) and three nonimmune cell types (epithelial cells, endothelial cells, and fibroblasts) (Figure 1B, Figure S1A, Table S2).

To better define the major population and subpopulation of the tumor‐infiltrating leukocytes, we then performed graph‐based cell clustering separately on these two datasets. For immune cells from the Smart‐seq2 platform, graph‐based cell clustering gave rise to 15 cell clusters (Figure 1D). T cells were the predominant immune cell type (49%), followed by B cells (39%), myeloid cells (6%), and mast cells (2%). An additional 4% of cells were positive for proliferation markers, which we annotated as cycling cells (Figure 1B). Each cluster contained cells from a number of different patients, indicating that cell types and expression states in the TME are largely consistent across CRC tumors and do not represent patient‐specific subpopulations or batch effects, although the cell‐type proportions do vary from person to person (Figure 1B). Not surprisingly, when comparing tumors and matching nontumor tissues, there were significant differences in the proportion of immune cells between tumor (CT and MT) and nonmalignant (ANT and DNT) tissues (Figure 1E,F). T cells and cycling cells were enriched in the tumor, whereas B cells and myeloid cells were depleted; mast cells were detected at similar proportions in both tissue types (Figure 1E,F and Figure S1E). In addition, the infiltration of T cells was significantly increased in both hepatic metastatic tumors and nontumor tissues (87.0% and 81.3%, respectively) (Figure 1E and Figure S1F), and the results were verified by flow cytometry (Figure S1G).

For the immune and nonimmune cells from the DNBelab C4 platform, graph‐based cell clustering also gave rise to 17 cell clusters (Figure S1B). Based on the expression of known markers, we found that the atlas mainly comprised epithelial cells (*EPCAM*, *CDH1*, *CD24*) and nonepithelial cells (Figure S1C). As shown in Figure 1D, the nonepithelial cell lineages comprised plasma cells (*JCHAIN*, *MZB1*), T cells (*CD3G*, *CD3D*), B cells (*MS4A1*, *CD79A*), fibroblasts (*DCN*, *COL1A2*), and endothelial cells (*GNG11*, *ENG*). The major immune‐cell populations, including T cells, B cells, and myeloid‐lineage cells for both platforms were qualitatively consistent, demonstrating the validity of our data (Figure S1D).

To gain deeper insights into tumor progression and tumor metastasis mechanisms in human CRC, we performed integrative analysis by using the full‐length scRNA‐seq sequences. The association analysis between tumor, node, and metastasis (TNM) staging and distinct cell type infiltration in CRC tumor revealed that infiltration of T cells was decreased in advanced stage CRC (stage III and IV), in contrast, mast cells were more enriched in advanced stage CRC (Figure 1G). This is consistent with a previous study that found that mast cells were recruited to cancer cells and released pro‐tumor factors to promote proliferation of cancer cells.^18^

Taken together, our results suggest that the proportion of cell subpopulations in tumor tissues is significantly different from that of paired normal tissues in CRC, and that liver metastases possess a distinct TME from that of primary CRC.

### 2.2 Heterogeneity and impairment of T cells provide new clinical insights

**FIGURE 2:**
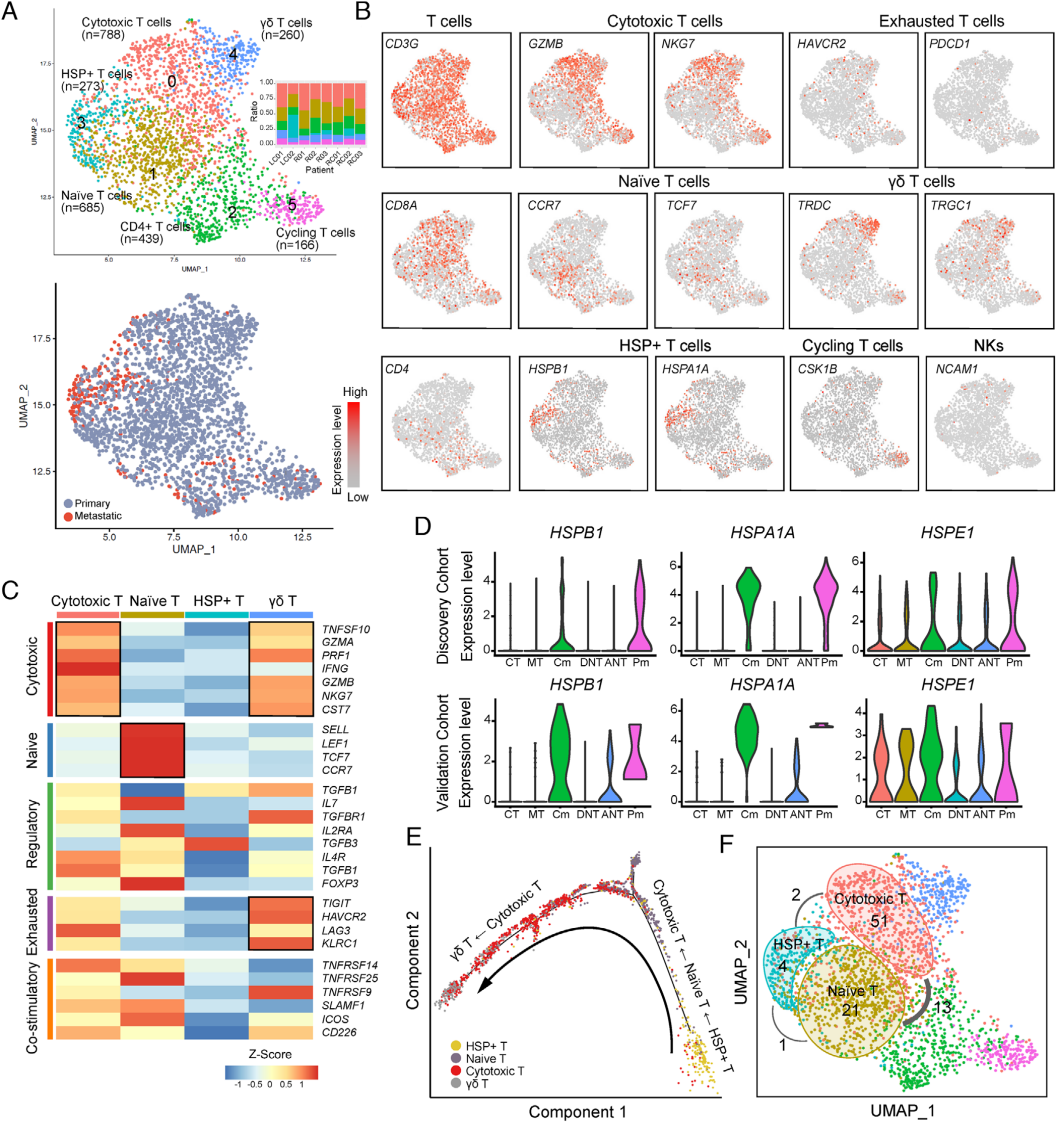
Characterization of T‐cell subpopulations in colorectal adenocarcinoma (CRC). A, Uniform Manifold Approximation and Projection algorithm (UMAP) plot of 2,571 T cells, color‐coded by their associated cluster and bar plot shows distribution of cell clusters among patients (top) or sample type (bottom). B, UMAP plot, color‐coded for the expression level (gray to red) of marker genes in each cell type. C, Heatmap showing the *z*‐score of T‐cell functional genes for each cluster. D, The expression level of heat‐shockprotein (HSP) genes in discovery cohort and validation cohort. E, The ordering of T cells along pseudotime in a two‐dimensional state‐space defined by Monocle2. Each point corresponds to a single cell, and each color represents a T‐cell cluster. F, Clone expansion and sharing among naïve, cytotoxic, and HSP^+^ T cells. The number within the circle refers to the expansion clone number in that specific cluster. Lines connecting clusters are based on the degree of TCR sharing, with the thickness of the line representing the number of shared TCR clones.

T cells in discovery cohort were further reclustered and yielded six distinct clusters (Figure 2A, Table S3). Cluster_0 cells, highly expressing *GZMB* and *NKG7*, were identified as cytotoxic T cells, which are associated with T‐cell activation and cytotoxic function (Figure 2B,C). Cluster_1 cells, expressing naïve T‐cell markers *CCR7* and *TCF7*, were characterized as naïve T cells (Figure 2B). Cluster_2 cells were identified as CD4^+^ T cells due to their expression of *CD4* and *CXCL13* (Figure S2A), which is a marker of exhausted CD4^+^ T helper cells.^19^ Cluster_3, namely HSP^+^ T cells, expressing a low level of both cytotoxic markers and naïve markers (Figure 2B,C), were distinct in the high expression level of heat‐shock protein (HSP) genes *HSPA1A*, *HSPB1*, and *HSPE1* (Figure S2A). Moreover, HSP^+^ T cells were more enriched in metastatic site than primary CRC (Figure 2A). Cluster_4 cells were identified as γδ T cells, as they specifically expressed *TRDC* and *TRGC* (Figure 2B); these cells also highly expressed cytotoxic markers *NKG7* and *GZMB*. Although the exhaustion markers *HAVCR2* and *PDCD1* showed low expression across our dataset, in this cluster the *HAVCR2* (TIM‐3) level was relatively higher (Figure 2C), which suggested that the γδ T cells possessed cytotoxic functions and partially showed the exhaustion state. Cells in Cluster_5 were classified as cycling T cells due to their signature expression of cell proliferation genes like *CKS1B* (Figure 2B and Figure S2A).

Surprisingly, the majority of Cluster_3 cells that expressed HSPs were derived from CLM, with 21.6% and 41.8% in metastatic site Cm and Pm, respectively. HSP‐related genes have been shown to be highly expressed in regulatory T cells (Treg) and CD4^+^ conventional T (Tconv) cells in breast carcinoma compared to normal breast parenchyma.^20^ Moreover, HSP70 has been shown to promote Treg survival and immune suppression.^21^ To exclude the possibility that the HSP^+^ T cells in our study might just be the artifact results of stress from cell sorting rather than a true state, we examined the expression of *HSPA1A*, *HSPB1*, and *HSPE1* in the validation cohort, and found a similar expression pattern of these HSP genes (Figure 2D). In addition, the presence of HSP^+^ T cells in Cm tissues was also confirmed by immunofluorescence (IF) using CD3 and HSP70 antibodies (Figure S2C). Therefore, we supposed these HSP^+^ T cells were in their true state. To elucidate whether the HSP^+^ T cells were more similar to the naïve state or the cytotoxic state, and their function, a trajectory analysis was performed on the naïve T cells, cytotoxic T cells, γδ T cells, and HSP^+^ T cells using Monocle2.^22^ As shown in Figure 2E, the trajectory began with the HSP^+^ T cells, followed by the naïve T cells, and then the cytotoxic T cells, and ended with the γδ T cells, suggesting that the HSP^+^ cells were more similar to the naïve T cells than the cytotoxic T cells, and with less activity than the naïve T cells. This might be due to the influences of the immune microenvironment in CLM.

As previously reported, the diversity of expanded T‐cell receptor (TCR) clones is associated with prognosis in tumor and a greater diversity of expanded TCR clones has been observed in activated T cells than in inactivated T cells.^23^ To further confirm the activity state of the HSP^+^ T cells, we assembled full‐length TCR α and β sequences for 938 T cells using TRACER,^24^ among which 84 clones were considered to be expanded clones consisting of more than two cells. We analyzed the clone expansion and clonal sharing between three αβ T‐cell subpopulations: naïve, cytotoxic, and HSP^+^ T cells. The number of expanded clones in these three T subtypes were 21, 51, and 4 (Figure 2F), respectively, along the trajectory path, indicating that the HSP^+^ T cells were more inactive than the naïve T cells. The results above suggest that the T cells from metastatic sites were less active with fewer expansion events than those from primary sites, demonstrating heterogeneity between the microenvironment of metastatic and primary sites.

**FIGURE 3:**
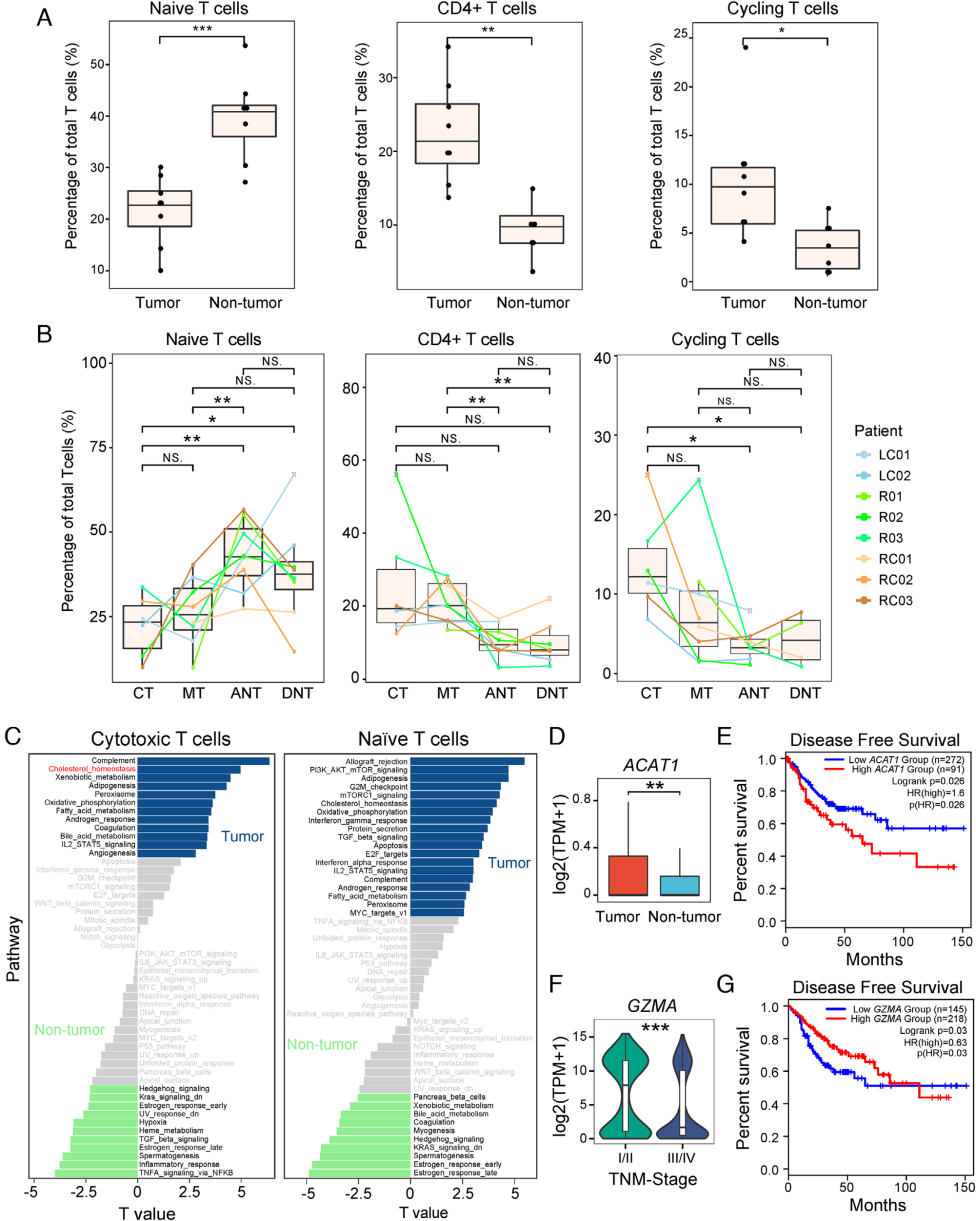
Interregion comparisons of T‐cell subpopulations. A, The percentage of cells in different T‐cell subpopulations in tumor and nontumor tissues. B, The percentage of cells in different T‐cell subpopulations in different dissection regions. C, Differences in pathway activities between tumor and nontumor T subtypes as scored per cell by GSVA. The *t*‐values from a linear model corrected for patient of origin are shown. D, The expression of *ACAT1* in cytotoxic T cells from tumor and nontumor tissues. E, Survival analysis based on the expression status of *ACAT1* (normalized by *CD3G*) in TCGA COAD and READ. F, *GZMA* was highly expressed in T cells from early CRC tumor. G, Survival analysis based on the expression status of *GZMA*. NS, *P* > 0.05; *P < 0.05; **P < 0.01; ***P < 0.001, as determined by Student's *t*‐test.

The difference between T cells in tumor tissue and nonmalignant tissue can also be observed, much as previously reported.^8^ For example, the naïve T cells were significantly enriched in the nonmalignant tissue (*P* < 0.001), while the *CD4* ^+^ T helper cells and cycling T cells were enriched in the tumor tissue (*P* = 0.0011 and 0.018, respectively) (Figure 3A). No significant difference was observed between the center and marginal regions of tumors, nor between adjacent noncancerous and distal noncancerous tissues (Figure 3B).

We further asked whether the same T‐cell subpopulations, but derived differently from tumor or normal tissues, had different gene expression patterns. We thus performed a gene set variation analysis (GSVA) for cells from tumor (CT and MT) and noncancerous (ANT and DNT) tissues, separately (see Section 4). For the cytotoxic T cells, tumor‐derived T cells showed stronger cholesterol homeostasis activities (Figure 3C) and significantly higher expression levels of *ACAT1*, a key cholesterol esterification enzyme (Figure 3D). This high level of cholesterol may be related to T cells dysfunction. According to Yang et al,^25^ the antitumor response of CD8^+^ T cells is associated with cholesterol metabolism in a skin melanoma mouse model. Inhibition of *ACAT1* enhanced the proliferation of CD8^+^ T cells and successfully delayed the growth of tumors in mice.^25^ It was also reported that the expression of *ACAT1* could serve as a potential prognostic marker in prostate cancer.^26^ To interrogate the association of *ACAT1* level with prognosis in CRC, we performed survival analysis and found that high *ACAT1* expression is related to poor disease‐free survival in colon adenocarcinoma and rectum adenocarcinoma cohorts from The Cancer Genome Atlas (TCGA) cohort (Figure 3E). Therefore, *ACAT1* may be a potential prognostic marker for CRC.

For the naïve T cells, the proportion of this cell type in tumors was significantly lower than in nontumor (Figure 3A), but strong proliferative activities (G2M checkpoint and E2F targets), allograft rejection activities, as well as strong IFN‐γ and IFN‐α responses were observed. This indicates that the naïve T cells in the TME may be more similar to activated T cells than the naïve T cells in adjacent normal tissues.

Finally, comparison of gene expression between early and advanced T cells revealed that the cytolytic molecule *GZMA* highly expressed in T cells derived from early CRC tumor (Figure 3F and Figure S2B). Upregulated *GZMA* in CRC (from TCGA) was also found to be associated with longer PFS (*P* = 0.036) (Figure 3G). Therefore, impairment of T‐cell‐meditated immune response and enrichment of mast cell could be a factor for poor prognosis of CRC.

Taken together, we observed the distinct T‐cell immune profiles of CRC tumor and normal tissues. Additionally, the same T‐cell subpopulations possessed different expression patterns in several pathways depending on their origins of either tumor or normal tissues.

### 2.3 Landscape of the heterogeneity and diversity of B cells in tumor microenvironment

**FIGURE 4:**
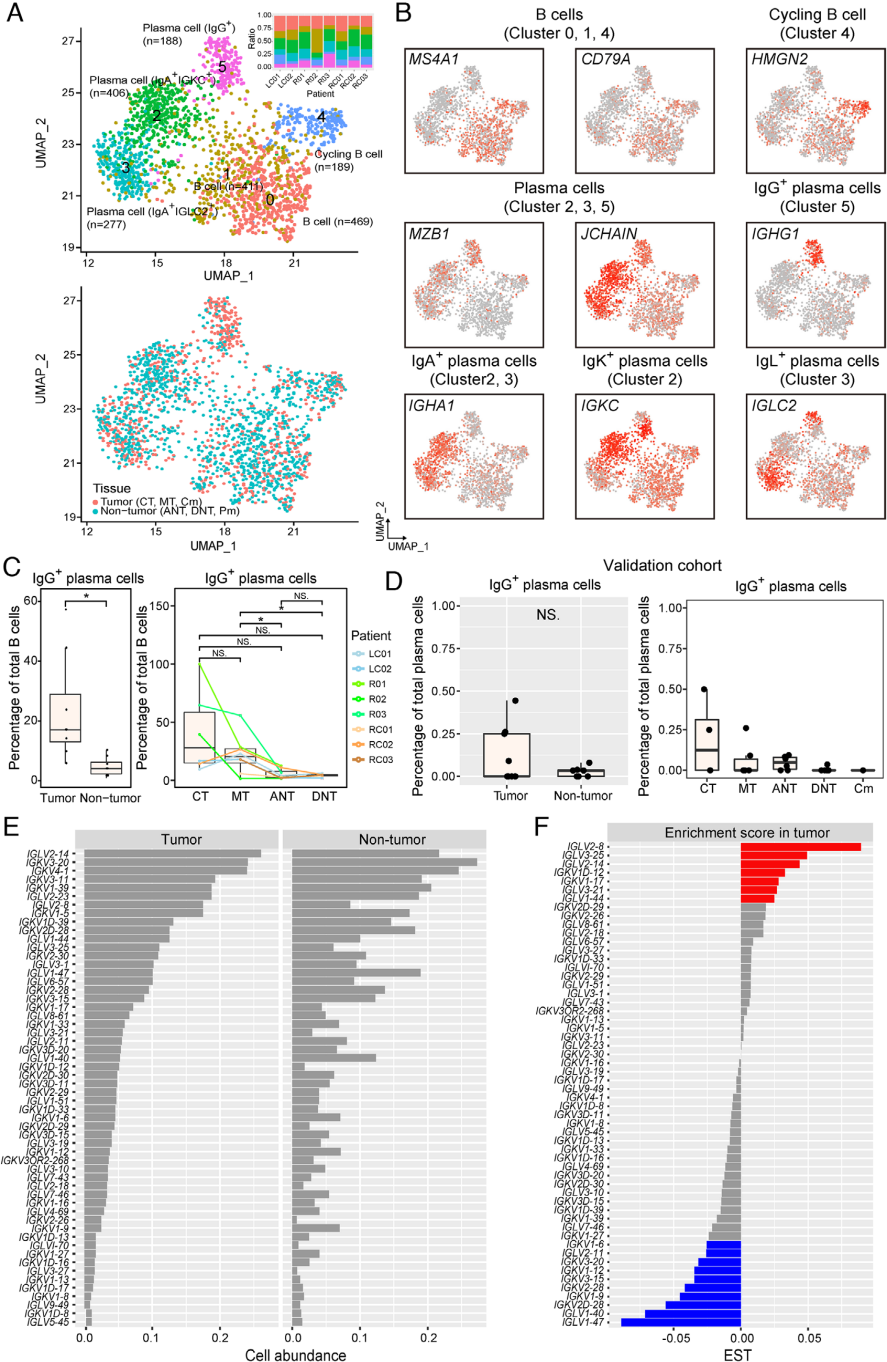
A distinctive landscape of B‐cell subpopulations in colorectal adenocarcinoma (CRC). A, Uniform Manifold Approximation and Projection algorithm (UMAP) plot of 1940 B cells, color‐coded by their associated cluster and bar plot shows distribution of cell clusters among patients (top) or sample tissue origin (bottom). B, UMAP plot, color‐coded for the expression level (gray to red) of marker genes in each cell type. C, The percentage of IgG plasma cells in tumor and nontumor tissues. D, The percentage of IgG plasma cells in tumor and nontumor tissues from validation cohort. E, Frequency of B cells expressing immunoglobulin (Ig) light chain variable genes in B‐cell population. Only 56 genes having expression across 20 B cells (1%) were selected to compare the frequency between tumor and nontumor region. F, The calculated enrichment score in tumor of 56 light chain variable genes in (E). Enrichment score in tumor = [frequency in tumor region] − [frequency in nontumor region]. NS, *P* > 0.05; *P < 0.05; **P < 0.01; ***P <0 .001, as determined by Student's *t*‐test.

In this study, a large number of B lymphocytes were found in tumor tissues, less than T lymphocytes, and accounted for 30.6% of all immune cells in primary CRC (Figure 1C). To further understand the heterogeneity of B cells and their immunological properties, we performed refined clustering of B cells, and identified three subpopulations of *MS4A1* ^+^ B cells and three plasma cell subsets (Figure 4A and Figure S3A, Table S4). Two of the B‐cell subsets were designated as tissue‐resident memory B cells as they showed the hallmark *CD69* and *CD44* expression (Figure S3B).^27^

Two of the plasma cell subpopulations (Cluster_2 and Cluster_3) highly expressed IgA‐related genes (IGHA1/2) (Figure 4A,B). Immunoglobulin κ constant (IGKC) was highly expressed in Cluster_2, also designated as IgA^+^IGKC^+^ cells. Cluster_3, also classified as IgA^+^IGLC2^+^ cells, were distinguished in the high expression of Ig lambda (IGL) genes (*IGLC2* and *IGLC3*), but low expression of *IGKC*. IgAs are a class of antibodies that are mainly distributed in mucosal areas, such as the gastrointestinal tract, respiratory tract, and genitourinary tract, and play an important role in preventing pathogen colonization.^28^ *IGKC* has been found to be associated with favorable prognosis in breast cancer, CRC, and non‐small cell lung cancer.^29^, ^30^ The third one, Cluster_5 plasma cells (IgG^+^), expressed immunoglobulin heavy constant gamma genes (*IGHG1‐4*), indicating that they specifically secreted IgG antibodies (Figure 4B). IgG^+^ plasma cells were significantly enriched in tumor tissues and their infiltration level gradually decreased along the center to the periphery of the tumor (Figure 4C). In the validation cohort, we also identified four plasma‐cell subtypes including IgA^+^IGKC^+^, IgA^+^IGLC2^+^, IgG^+^, and IgM^+^ plasma cells, and a group of plasma cells with unique profile of immunoglobulin variable region genes, which resided only in normal tissue from an individual patient (Figure S4A,B). The infiltration of IgG^+^ plasma cells was also found to be higher in tumor in validation cohort (Figure 4D), but the difference was insignificant. This may be due to the biases associated with the two platforms (Smart‐seq2 and DNBelab C4), which led to loss in certain cell populations in validation cohort. Consistent with the results in the discovery and validation cohorts, FACS analysis showed the abundance of IgG^+^ plasma cells in the CRC tumor tissues (Figure S4C).

B‐cell receptor (BCR) diversity of infiltrating B cells is important for TME. It has been previously reported that in some cancers the BCR light chain diversity of infiltrating B cells in the tumor region was significantly higher than the B cells in the nontumor region.^31^ Interestingly in our study, the BCR light chain showed high diversity and abundance in both tumor and nontumor tissues; however, these two tissues did not differ significantly from each other (Figure 4E). This can be explained by the fact that the immune system of the gastrointestinal mucosa is influenced or complicated by antigens from the gastrointestinal microbiota and food intake.^15^, ^16^ We next asked whether there were specific types of BCR genes expressed only in the tumor tissue of CRC. Although most of the light chain variable region genes (IGLV genes and IGKV genes) were not uniquely expressed in B cells in the tumor region, the abundance of each variable region genes in tumor and nontumor regions was indeed different (Figure 4F). The variable region genes that showed significant differences in abundance between tumor and normal regions, such as *IGLV2‐8*, *IGLV3‐25*, and *IGLV2‐14* (Figure 4F), might be associated with antitumor antibodies or with disordered gastrointestinal microbiota in CRC.^32^

In summary, we provided a landscape of B lymphocytes in CRC, and found the different cell components in different regions. Furthermore, we found a set of BCR light chain variable region genes expressed differentially in the tumor region, which should help to gain deeper insight into the humoral immunity and the relationship between gastrointestinal microbiota and tumor progress for CRC.

### 2.4 Various B‐cell subtypes execute different antitumor responses in CRC

**FIGURE 5:**
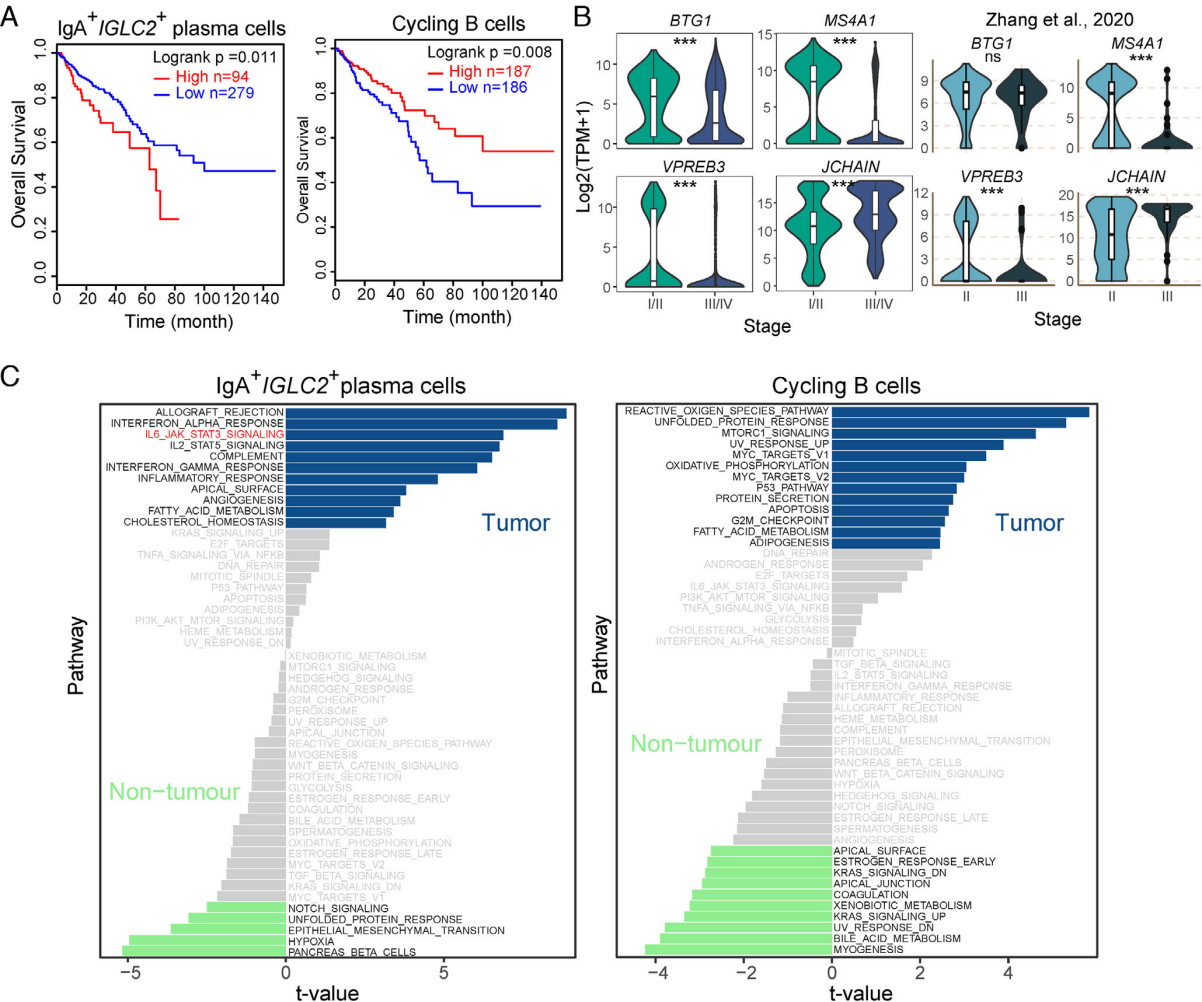
Characterization of B‐cell subpopulations in colorectal adenocarcinoma (CRC). A, The Kaplan‐Meier overall survival curves of TCGA CRC patients grouped by the mean expression of feature of IgA^+^IGLC2^+^ plasma cells and cycling B cells. B, Violin plot comparing the gene expression of B cells from early and advanced CRC tumors from discovery cohort (left panel) and Zhang et al. (right panel). NS, *P* > 0.05; *P < 0.05; **P < 0.01; ***P < 0.001, as determined by Student's *t*‐test. C, Differences in pathway activities between tumor and nontumor B‐cell subtypes as scored per cell by GSVA. The *t*‐values from a linear model corrected for patient of origin are shown; dn, down; UV, ultraviolet; v1, version 1; v2, version 2.

Infiltrating B cells are commonly observed in a variety of tumor tissues,^33^, ^34^, ^35^ yet their reported correlation with patient outcomes has been inconsistent.^34^, ^36^, ^37^ For example, the infiltration of *MS4A1* ^+^ B cells and plasma cells in tumor lesion was related to favorable prognosis in breast cancer, non‐small cell lung carcinoma, melanoma, and CRC.^30^, ^38^, ^39^, ^40^ However, other studies have shown that increased infiltration of *MS4A1* ^+^ B and plasma cells is associated with poor prognosis in epithelial ovarian cancer and invasive ductal breast cancer^41^, ^42^ We hypothesized that this inconsistency may be due to the presence of distinct B‐cell subpopulations performing distinct biological functions masked by bulk analysis. To reveal the association of different B‐cell subtypes with CRC prognosis, the features of B‐cell subtypes were quantified, and the relationship between these features and the CRC prognosis was analyzed in a CRC cohort from TCGA. The results showed that CRC patients with higher levels of IgA^+^IGLC2^+^ plasma cells tended to have poor prognoses (Figure 5A and Figure S3D). Conversely, high cycling B‐cell (Cluster_4) scores were associated with prolonged survival. These results indicate that B‐cell subpopulations may indeed have distinct roles in the antitumor response in CRC.

We next compared the expression profiles of tumor‐derived and normal tissue‐derived B‐cell subsets IgA^+^IGLC2^+^ plasma and cycling B cells. For IgA^+^IGLC2^+^ plasma cells, allograft rejection, interferon‐alpha response, interferon‐gamma response, and inflammatory response were enhanced in the tumor‐derived cells, indicating the involvement in promoting inflammatory responses in tumors. However, angiogenesis and activation of IL‐6‐mediated JAK/STAT3 signaling was also observed in these cells. According to a previous study, activation of angiogenesis in tumor‐derived cells has been shown to be associated with poor CRC prognosis.^43^ In addition, The IL‐6‐mediated JAK/STAT3 signaling pathway is closely related to the formation of inflammatory bowel disease and diverse human solid tumors, including CRC.^47^, ^48^ For cycling B cells, stress‐related responses including reactive oxygen species pathways, unfolded protein responses, and ultraviolet responses were activated in the tumor‐derived cells, and so was the enhancement of cell proliferation‐related pathways (Figure 5C).

To explore the association between TNM‐stage and B‐cell activity, we conducted differential expression analysis for B cells from early and advanced CRC tumors (Figure S3E). An antiproliferative protein gene *BTG1* was highly expressed in infiltrating B cells of the early CRC tumor (Figure 5B). Pre‐B lymphocyte protein 3 gene (*VPREB3*), which is thought to be involved in B‐cell maturation and development,^41^ was also upregulated in early CRC tumor (Figure 5B). Moreover, the B‐cell marker *MS4A1* was upregulated, and the plasma cell marker *JCHAIN* was downregulated in early CRC tumor (Figure 5B). *TCL1A* and *BTG1*, which are highly expressed in B cells from early CRC tumor (Figure 5B and Figure S3E), are reported as tumor suppressors in cervical cancer and CRC, respectively^44^, ^45^, ^46^. In addition, plasma cells were more abundant in advanced CRC and vice versa (Figure S3C). These results indicate that B cells from early CRC tumors are pre‐B like cells and may have antitumor capabilities, while B cells from advanced CRC tumors fully develop into plasma cells.

Taken together, these analyses of the B‐cell subpopulations in CRC expand our understanding of B‐cell activities during the progression of CRC. Prognostic correlation analysis revealed a link between two B‐cell subsets and prognosis, which provides a useful clue for the clinical treatment of CRC.

### 2.5 Transitions in cell‐cell interactions in the CRC microenvironment reshape immune capability

**FIGURE 6:**
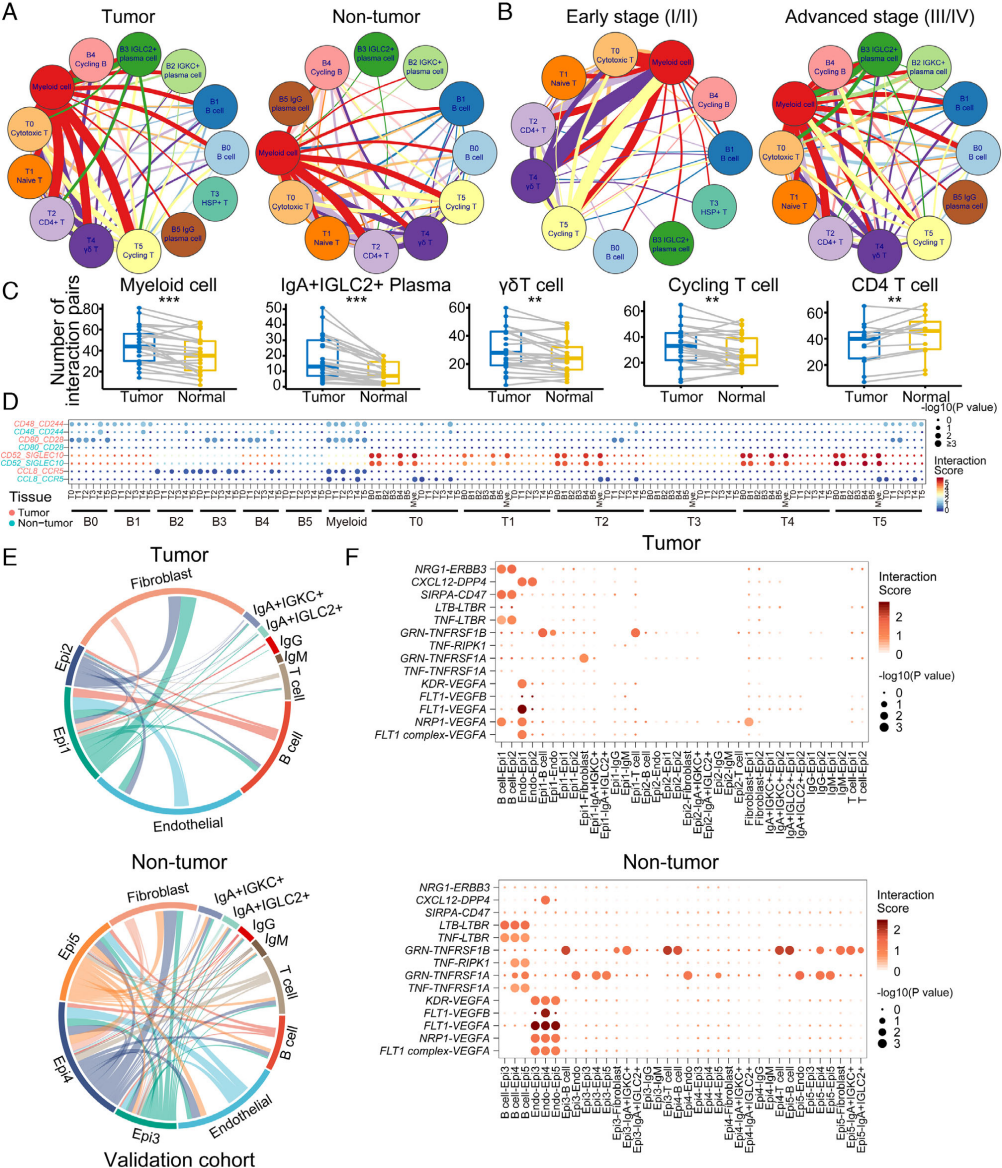
Cell‐cell communication in the colorectal adenocarcinoma (CRC) tumor microenvironment. A and B, Network of potential cell interactions in tumor and normal tissues (A) as well as in early and advanced CRC tumors (B). Width of lines indicates the number of ligand‐receptor pairs between the indicated cell types (interactions with fewer than 20 ligand‐receptor pairs are not shown). C, The difference of number of interaction pairs in different cell types between tumor and nontumor. NS, *P* > 0.05; *P < 0.05; **P < 0.01; ***P < 0.001, as determined by Student's *t*‐test. D, Overview of selected ligand‐receptor interaction pairs in tumor and nontumor tissues. *P*‐value is indicated by the size of the circle; color indicates the interaction score, which refers to the mean total of all individual ligand‐receptor partner average expression values. E, Network of potential interactions between epithelial cells and immune cells or stoma cells in validation cohort. Width of lines indicates the number of ligand‐receptor pairs between the indicated cell types. F, Overview of selected ligand‐receptor interaction pairs in tumor and nontumor tissues in validation cohort.

The immune microenvironment formed by the interaction between immune cells affects the occurrence and progression of tumor cells.^43^ We used CellPhoneDB^47^ to analyze the interaction between immune cells in CRC (See Section 4). By comparing the interaction network between tumor tissues and nontumor tissues, we found that the communication of myeloid cells with other immune cells was significantly increased in tumor tissues (Figure 6A,C). This suggests that myeloid cells may act as hub for regulating the immune responses in CRC tumors. Interactions of IgA^+^IGLC2^+^ plasma cells, γδ T cells, and cycling T with other immune cells also increased, while interactions of the CD4^+^ T cells with the myeloid cells and most T cells decreased (Figure 6A,C). It can, therefore, be hypothesized that immunoregulatory functions in CRC primarily depend on the myeloid cells and B cells rather than on the CD4^+^ regulatory T cells. Comparing cell‐cell interactions for early and advanced CRC tumors indicates that cell‐cell interactions were more complex, and the B lymphocytes interaction increased in advanced CRC tumors (Figure 6B). Notably, IgA^+^IGLC2^+^ plasma cells, which were associated with poor prognosis in CRC, showed a significant interaction with the myeloid cells and cytotoxic T cells in advanced CRC tumors.

TILs sense tumor antigens and are activated by antigen‐presenting cells to become the primary final effectors of tumor cell death.^48^ The ability of T cells to fight tumors depends on their invasive site and the balance between costimulation and immune suppression.^49^ To reveal how the state of T cells is regulated by other immune cells in CRC, we evaluated the expression of immune response, immune stimulation, inhibition, and chemokine‐related genes of T cells in tumor and adjacent normal tissues, as well as the interactions between T cells and other immune cells (Figure S5A). In CRC tumor infiltration sites, CD28‐CD80 costimulation signaling was observed in the myeloid cell‐T cell interactions (Figure 6D). Interestingly, CD48‐CD244 interactions were also enriched in the myeloid cell‐T cell and B cell (B0 and B1)‐T cell interactions. Previous studies have shown that CD244 is associated with T‐cell exhaustion in tumors.^50^ CD52 is a regulatory molecule that binds to SIGLEC10 and inhibits T‐cell activation.^51^ In our study, B cells (B0, B1, and B4) and myeloid cells expressing CD52 tended to interact with and inhibit various SIGLEC10^+^ T cells (Figure 6D). The CCL8/CCR5 signaling axis is known to recruit CCR5^+^ T cells.^52^ In our study, IGLC2^+^ plasma cells and cycling B cells (B3 and B4) expressing CCL8 interacted with CCR5^+^ T cells in CRC (Figure 6D). These results indicate that the myeloid cells exhibit both proimmune and immunosuppressive activities in CRC, and the IGLC2^+^ plasma cells and cycling B cells in CRC can recruit CCR5^+^ T cells to tumor lesions.

Moreover, nonimmune cells were obtained in validation cohort. To further investigate cell‐cell interactions between immune/stoma cell and epithelial cells, we inferred the interactions between epithelial cells and immune cells as well as stroma cells. First of all, we grouped epithelial cells into five subtypes according to cell distribution in sampling site and their transcriptome profile (Figure S5B‐D). Epi1 and Epi2, mainly from tumor tissue, show lower expression of metallothionein genes (Figure S5C). Previous study reported that metallothionein genes were downregulated in CRC tumor tissue.^53^ Epi1 consisting of cluster 2 and 4 of epithelia also showed higher cell cycle, epithelial‐mesenchymal transition (EMT), invasion, and metastasis score (Figure S5D), which indicated that Epi1 may be malignant cell. In tumor microenvironment, epithelial cells show fewer potential interactions with immune cells compared with normal tissue (Figure 6D). We found that endothelial cells from normal tissue would receive stronger angiogenic stimulatory signals from epithelia cells through VEGF and its receptor FLT1/NRP1/KDR. Epithelia cells in tumor show a lower activation of tumor necrosis factor signaling from B cells. Several interaction pairs were found only between epithelial cells and B cells in tumor tissue, including SIRPA‐CD47 and NRG1‐ERBB3 (Figure 6E), which were related to immune escape and EMT‐related metastasis. In total, epithelial cells may regulate endothelial cells to form the microvessel in nontumor tissue, and B cells in CRC may contribute to progress of tumor.

Taken together, these results suggest that myeloid cells and B cells play a critical role in CRC immune regulation. The antitumor response of T cells in CRC tumors was activated and conversely attenuated by myeloid cells in the TME. This reflects “accelerator” and “brake” mechanisms of the immune system in CRC.

## 3 DISCUSSION

It has been widely accepted that TME, and in particular its immune response, is crucial for the modulation of tumor progression and responsiveness to therapy.^4^ Here, we present a comprehensive single‐cell transcriptomic atlas of 5,345 immune cells isolated from multiple sites of eight CRC patients, providing a rich resource for understanding the multidimensional characterization of immune cells in CRC.

T and B lymphocytes are the predominant infiltrating cell types in the CRC immune ecosystem. In this study, we focused on the heterogeneity between different sampling sites. The distribution of different types of TILs varied between tumor and normal tissues. In particular, the proportion of the naïve T cells in tumor tissues was significantly lower than in normal tissues, whereas the cycling T cells exhibited the opposite distribution pattern, indicating that T cells in tumor tissues were more likely to be activated and be in the proliferation stage. Hepatic metastasis is the dominant metastatic site for patients with CRC.^54^ We documented that the T cells in hepatic metastatic sites were highly abundant, considering the limitation of sample size, we validated the existence of HSP^+^ T cells by immunofluorescence (Figure S2C). In addition, these HSP^+^ T cells were characterized by the naïve and inactive features, as well as HSP genes, which were reported to be related to T‐cell suppression in many studies.^21^, ^55^ Stimulating these hepatic metastasis‐derived T cells from their naïve state to the activated state may be a potential strategy to treat CRC‐associated liver metastatic disease. Also, in the present study, the tumor‐derived cytotoxic T cells showed significantly enhanced expression of cholesterol homeostasis‐related genes and higher expression of *ACAT1*. Therefore, blocking cholesterol synthesis enzymes (such as ACAT1) in the T cells in tumor tissues may facilitate treatment of CRC.

B cells are a dominant immune cell type in CRC that up to now have only been studied to a very limited extent, and with incompatible opinions on their role in tumor progression. In this study, we for the first time identified six subtypes of B cells in CRC and provided a comprehensive transcriptomic profile, and we suggested that the previous conflicting results on B‐cell effects on tumors may be due to the diversity in the roles for the B‐cell subtypes. Importantly, we identified *MS4A1* ^+^ B cells and plasma cells that expressed different Ig isotypes (IgA and IgG). IgG‐secreting plasma cells were dominant in the tumor center and decreased progressively from the tumor center to normal adjacent tissue. Previous studies have shown that higher levels of ex vivo IgG responses to tumor‐associated antigens were related to shorter recurrence‐free survival (RFS) in breast cancer, whereas IgA levels were not significantly associated with RFS.^56^ Beyond this, several studies have demonstrated that IgG was expressed in various cancer cell types and was involved in the development and growth of tumor cells including in CRC, LNCaP prostate cancer cells, breast, liver, and lung cancers.^57^, ^58^, ^59^ These results imply that the blockage of IgG might be a targeted therapy of CRC. Two B‐cell subpopulations (IgA^+^IGLC2^+^ plasma cells and cycling B cells) were found to have opposite effects on CRC prognosis, as the former correlated with poor patient survival, whereas the latter correlated with favorable patient survival. According to cell‐cell communication analysis, the highly proliferative*IGLC2* ^+^ plasma cells and cycling B cells were associated with a better prognosis and may recruit *CCR5* ^+^ T cells via CCL8 in CRC tumors.

As for the myeloid cells, these cells were shown to be involved in T‐cell exhaustion and activation through different ligand‐receptor interactions, such as CD48‐CD244 and CD28‐CD80. This may also be attributed to the heterogeneity of myeloid cells. As for the mast cells, we found that they accumulated more in advanced CRC tumors, which supports the previous finding that mast cells promote CRC growth.^18^ However, the role of mast cells and their interaction with colon cancer cells is still poorly understood. Further study on mast cells will contribute to our understanding of its function and immune mechanism in tumors.

Using the validation cohort, we elucidated the intercellular interactions between epithelial cells and immune and other stroma cells in the TME of CRC. Angiogenic stimulatory signals were found to be higher in endothelial cells from normal tissue, which indicates that vascular formed around tumor may facilitate metastasis of tumor cells. The SIRPA‐CD47, CXCL12‐DPP4, and *NRG1‐ERBB3* interactions were found between *MS4A1* ^+^ B cells and epithelial cells in tumor. It has been suggested that blockade of the SIRPα‐CD47 checkpoint may provide a potential new way to treat cancer.^60^ In addition, CXCL12 inhibits DPP4 and accelerates EMT and metastasis in breast cancer,^41^ and the inhibition of ERBB3 signaling suppresses EMT of hepatocellular carcinoma.^42^ The B cells in CRC may also facilitate immune escape and metastasis of tumor, and this provides a potential target for the treatment of CRC.

Our study suggests that the antibody‐producing cells in TME of CRC were mainly B cells and plasma cells. This is consistent with increasing evidences that link the gastrointestinal microbiota and the CRC progression.^61^, ^62^, ^63^ Our single‐cell analyses provide a new perspective for the relationship, as we profiled the BCR in both tumor and nontumor regions for CRC patients. While we are unable to assign specific BCRs to the antigens from gastrointestinal microbiota, it is reasonable to speculate that the TME of CRC is coconstructed by gastrointestinal microbiota and tumor. The relationship between the immune repertoire of CRC and the gastrointestinal microbiota needs to be further explored, which is essential for the development of novel immune therapies for CRC.

In summary, our study highlights the diverse phenotypes of immune cells, in particular the B cells, in the CRC microenvironment. This immune‐cell atlas adds to the resources for identifying clinically relevant predictive markers for immunotherapy.

## 4 METHODS

### 4.1 CRC patients and tissue samples

This study was approved by the local ethics committee of Guangdong Academy of Medical Sciences and General Hospital (License No. 2017233H [R2]) and complied with all relevant ethical regulations. All patients signed informed consent forms before recruitment.

Fourteen male and four female patients who were pathologically diagnosed with CRC at the Guangdong Academy of Medical Sciences and General Hospital were enrolled in this study. Their ages ranged from 30 to 83 years, with a median age of 61. Among these patients, two were diagnosed at stage I, four at stage II, nine at stage III, and four at stage IV. One patient (NR01) with rectal cancer had received neoadjuvant chemoradiation therapy before tumor resection, all other patients had not received radiotherapy or chemotherapy prior to tumor resection. The available clinical characteristics of these patients are summarized in Table S1.

Human tissue specimens were collected at the time of surgery resection under the supervision of a qualified pathologist. For each patient, three types of fresh tissues were collected during the operation, including primary tumor tissue, adjacent noncancerous tissue (to the brim of matched tumor 3‐5 cm), and DNT (to the brim of matched tumor ≥10 cm). Then, two representative areas of the tumor center (CT) and the invasive margin (MT) were selected and cut from the primary tumor tissue. For patient LC02 and NLC08, liver metastases (Cm) and adjacent noncancerous tissues (Pm, to the brim of matched tumor 3 cm) of the liver were also collected. All collected samples were kept on ice‐cold RPMI‐1640 medium (Invitrogen) before processing.

### 4.2 Preparation of single‐cell suspensions

Two methods were used to prepare single cell suspension.

For patients RC01, RC02, RC03, R01, R02, R03, LC01, LC02, NR01, NLC03, and NLC04, cancerous tissues and noncancerous tissues, collected from the primary surgical or liver metastatic specimens, were mechanically and enzymatically disaggregated into single‐cell suspensions, following previously published methods.^64^, ^65^ Briefly, after the tissue dissection, each tissue was washed with HPBS twice and cut into small pieces (0.2‐0.5 mm^3^) with scissors, resuspended in RPMI‐1640 medium (Invitrogen) containing 10% fetal bovine serum (FBS; GIBCO) and digested into single‐cell suspensions with 1 mg/mL type I collagenase (GIBCO, #17100017) and 0.5 mg/mL type IV collagenase (GIBCO, #17104019) at 37°C for 2 hours, flick every 30 minutes. After dissociation, cell suspensions were serially filtered with a 40 μm cell strainer (BD) and centrifuged at 500 × *g* for 5 minutes, the supernatant was discarded and the cell pellet was resuspended with 1 mL of freezing buffer (90% FBS with 10% DMSO). Cells were then transferred into a Nalgene Mr Frosty Cryo 1°C freezing container (Thermo Fisher Scientific, #5100‐0001) and placed at −80°C for 12 hours before being transferred to liquid nitrogen for storage.

For the other patients, cancerous tissues and noncancerous tissues were separately cut into approximately 1‐2 mm^3^ pieces in the DMEM medium (Biosharp) with 10% FBS (GIBCO), and enzymatically digested with MACS tumor dissociation kit (Miltenyi Biotec) for 30 minutes on a rotor at 37°C, according to manufacturer's instruction. After dissociation, cell suspensions were serially filtered with a 40 μm cell strainer (BD) and centrifuged at 400 × *g* for 8 minutes, then the supernatant was discarded and the cell pellet was resuspended in red blood cell lysis buffer (Solarbio) and incubated on ice for 2 minutes to lyse red blood cells. After washing twice with 1× PBS (Invitrogen), the cell pellets were resuspended in 2 mL 1× PBS containing 0.04% bovine serum albumin (Sangon Biotech, A600903). Quantification of cell yields was performed by both Trypan blue dye exclusion staining counted with a hemocytometer and a handheld automated cell counter based on the Coulter principle (Scepter 2.0, Millipore). The final cell concentration was adjusted to 1,000 cells/μL for single‐cell library preparation.

### 4.3 Single‐cell RNA‐seq process for CRC patients (Smart‐seq2)

For Patient RC01, RC02, RC03, R01, R02, R03, LC01, and LC02, single‐cell RNA‐seq were performed by the Smart‐seq2 platform. The cryopreserved single‐cell suspensions from the above eight patients were placed in a 37°C water bath for rapid recovery and washed twice with 1× PBS, then single‐cell suspensions were stained with antibodies against CD45 (antihuman CD45, BD Biosciences) for FACS sorting. Single cells of different subtypes of immune cells were sorted into 72 × 72‐microwell chips (WaferGen Biosystems) with lysis buffer (10% Triton X‐100 0.5 nL, 40 U/μL RNase inhibitor 1.25 nL, 10 μM Oligo‐dT primer 12.5 nL, 10 mM dNTP mix 12.5 nL, and spike‐in RNAs 10 nL, nuclease‐free water 13.25 nL) dispensed into every microwell on the chip.

We prepared single‐cell transcriptome amplifications by the MIRALCS method following a modified Smart‐seq2 protocol.^66^ The External RNA Controls Consortium (ERCC) spike‐in mRNAs (1:50,000 , Ambion, Life Technologies) were added into the lysis buffer as the exogenous spike‐in control before the reverse transcription. We quantified the amplified cDNA products of each single cell by SmartChip real‐time PCR with the values of cycle threshold, melting temperature, and Agilent Bioanalyzer 2100. The single‐cell samples with high quality after amplification were extracted by an automatic extractor from the chip to a 96‐well plate and then used for library construction. We prepared the libraries according to the MIRALCS method,^66^ but cyclized the libraries into ssDNA libraries at the last step. Each single‐cell sample was labeled with a barcode. All single‐cell samples were sequenced by a BGISEQ500 sequencer with 100‐bp single‐end reads.

### 4.4 Single‐cell RNA‐seq process for CRC patients (DNBelab C4)

For Patients NRC01, NRC02, NR01, NLC03, NLC04, NLC05, NLC06, NLC07, NLC08, NLC09, and NLC10, single‐cell RNA‐seq were performed by the DNBelab C4 platform. The DNBelab C Series Single‐Cell Library Prep Set (MGI, #1000021082) was utilized as previously described.^17^ In brief, single‐cell suspensions were used for droplet generation, emulsion breakage, beads collection, reverse transcription, and cDNA amplification to generate barcoded libraries. Indexed single‐cell RNA‐seq libraries were constructed according to the manufacturer's protocol. The sequencing libraries were quantified by QubitTM ssDNA Assay Kit (Thermo Fisher Scientific, #Q10212). DNA nanoballs (DNBs) were loaded into the patterned nano arrays and sequenced on the ultra‐high‐throughput DIPSEQ T1 sequencer using the following read length: 30 bp for read 1, inclusive of 10 bp cell barcode 1, 10 bp cell barcode 2, and 10 bp unique molecular identifier (UMI), 100 bp of transcript sequence for read 2, and 10 bp for sample index.

### 4.5 Comparing IgG^+^ plasma cells from different tissue sites by flow cytometry

Single cells from fresh primary tumors, adjacent noncancerous tissue, and distal noncancerous tissue were obtained. After blocking with Fc Receptor Blocking Solution (Biolegend Cat #422301), cell surface staining was performed in FACS buffer containing antibody cocktails (anti‐CD45, anti‐CD38, anti‐CD19) on ice for 1 hour. For detection of total IgG, cells were stained for both surface IgG and intracellular IgG. The intracellular IgG was strained by using an Intracellular Fixation and Permeabilization Buffer Set (BD Biosciences Cat #558126) according to the manufacturer's protocols.

### 4.6 HE and IF staining of hepatic metastatic tumor in CRC patients

Human tissue specimens were provided by Guangdong Academy of Medical Sciences and General Hospital. The specimens were collected within 30 minutes after the tumor resection and fixed in formalin for 48 hours. Dehydration and embedding in paraffin was performed following routine methods. Specimen slices were cut from paraffin‐embedded tissue blocks with a microtome, deparaffinized and rehydrated. For HE staining, the slices were sequentially immersed in HE. For IF staining, the slices were further processed, including antigen retrieval, blocking of endogenous peroxidase, primary antibody incubation (included CD3 epsilon antibody [1:200, Affinit #DF6594] and HSP70 antibody [1:200, Affinit #AF5466]), secondary antibody incubation (included Alexa Fluor 647‐labeled goat anti‐rabbit IgG [H+L], [1:200, Beyotime #A0468], and FITC‐labeled goat anti‐rabbit IgG [H+L] [1:200, Beyotime #A0562]), and nuclear staining (DAPI). Finally, the slices were sealed with mounting medium for imaging. Images were taken on a LSM800 (Carl Zeiss) confocal microscope with 100× oil immersion lens.

### 4.7 Acquisition of single‐cell gene expression matrices and cell clustering

Raw reads were cleaned using Cutadapt (Version 1.15) and mapped to hg38 using STAR (Version 020201). Gene expression levels of each cell were quantified using RSEM (Version 1.3.0) and combined in R (Version 3.5.1). Cell clustering was accomplished by the Seurat R package (Version 3.0.1).^67^ Cells with less than 500 genes (TPM > 1) or over 20% TPM derived from the mitochondrial genome were removed, after which 5345 cells remained. Only protein‐coding, TCR, and IG genes were used for clustering, and genes expressed (TPM > 1) in less than three cells were discarded. TPM matrices from each patient were log‐normalized, and 2,000 variably expressed genes were selected using Seurat's FindVariableFeatures function. To remove batch effects, TPM matrices of different patients were integrated by Anchors, using the FindIntegrationAnchors and the IntegrateData functions from the Seurat R package. To reduce dimensionality, variably expressed genes were summarized by principle component analysis. The *t*‐distributed Stochastic Neighbor Embedding algorithm loses global structure due to a focus on local information,^68^ and as such we chose the Uniform Manifold Approximation and Projection (UMAP) algorithm to visualize the data.^69^ The top 10 principle components (PCs) were used as input to the RunUMAP function with default settings. The top 10 PCs were used as inputs in the FindClusters function with the “resolution” parameter set to 1.0 to find clusters. Cell clusters in the resulting two‐dimensional representation were annotated to known biological cell types using canonical marker genes (Figure S1A). Of note, very few lymphocytes (∼4%) were positive for cell proliferation markers. We therefore opted not to correct our gene expression matrices for effects of cell cycle. To better identify T‐cell and B‐cell subtypes, we extracted T cells and B cells and ran Seurat, respectively. Parameters were the same as introduced previously, except resolution was adjusted to 0.6 for T cells.

For DNBelab C4 data, raw sequencing reads were filtered and demultiplexed using PISA (Version 439 0.2; https://github.com/shiquan/PISA). The filtered reads were aligned to hg38 genome using STAR and sorted by sambamba (Version 441 0.7.0). Cell versus gene UMI count matrix was generated using PISA. Cells with less than 500 genes (UMI > 1) or over 25% UMI derived from the mitochondrial genome were removed, after which 9,770 cells remained. The clustering parameter was similar to Smart‐seq data, with PC usage set to 20.

We used harmony (https://github.com/immunogenomics/harmony) to integrate the data from two platforms with 20 PCs.

### 4.8 Identifying marker genes

We used the Seurat's FindAllMarkers function to identify marker genes for each cluster. This function contrasted cells from each cluster to all other cells of that cluster. The “RNA” assay and the “Wilcox” test were used for the function. Marker genes found by the FindAllMarkers function were required to have an average expression in that cluster that was >1.5‐fold higher than the average expression in the other clusters, and a detectable expression in >15% of cells from that cluster.

Marker genes of main cell types (Table S2), T‐cell subtypes (Table S3), and B‐cell subtypes (Table S4) were identified separately as described above.

### 4.9 Cell developmental trajectory

Cell lineage trajectory of T cells was inferred by using Monocle2.^22^ We first used the “relative2abs” function in Monocle to convert TPM into normalized mRNA counts and created an object with parameter “expressionFamily = negbinomial.size” following the Monocle2 tutorial. We used the “differentialGeneTest” function to derive differentially expressed genes from each cluster and genes with *q*‐value < 1*e*‐5were used to order the cells in a pseudotime analysis. After the cell trajectories were constructed, differentially expressed genes along the pseudotime were detected using the “differentialGeneTest” function.

### 4.10 TCR analysis

We used the TraCeR^70^ method to assemble TCR sequences for T cells. TraCeR can identify rearranged TCR chains and calculate their TPM values. After assembly, we arranged the productive TCR chains of every T cell by their TPM values. For example, if two TCRα chains were assembled in one single cell and were both productive, the chain with the higher TPM was defined as TCRα1, while the chain with the lower TPM being defined as TCRα2. Nonproductive TCR chains were excluded. The same arrangement was deployed on TCRβ. We kept cells with at least one pair of productive TCRα and TCRβ chain for subsequent analysis. We used a strict standard to define TCR clones: cells with the same TCRα1 and TCRβ1 were considered to be one TCR clone, while the expanded clones were defined as clones with at least two cells sharing the same TCRα1 and TCRβ1 in a given cell population.

### 4.11 GSVA and differential activities of pathways

Pathway analyses were predominantly performed on the 50 hallmark pathways described in the molecular signature database. To assign pathway activity estimates to individual cells, we applied GSVA^71^ using standard settings, as implemented in the GSVA package (Version 1.30.0). Differential activated pathways between cells from tumor and nontumor tissues were tested using the generalized linear model from the Limma package (Version 3.38.3), and *P*‐values were Benjamini‐Hochberg‐corrected. Pathways with adjusted *P*‐value < .05 were considered to be significantly differentially activated. Tumor features, cell cycle, EMT, invasion, and metastasis, were quantified using genes involved in these pathways by GSVA (Table S5).

### 4.12 Survival analysis

The TCGA provisional data were used to evaluate the prognostic effect of gene sets derived from specific cell clusters. The provisional gene expression and survival data of the TCGA were accessed using CBioPortal. We used Limma to perform differential expression gene analysis for each cluster and defined genes with more than two‐fold increase in expression in one cluster compared to the other clusters as “signature genes.” For the signature gene sets of each cluster, we calculated a “gene signature score” for each patient using fold‐change values for each gene in the signature to weigh genes in calculations of the average. Then, patients were grouped into high‐ and low‐expression groups by the median value of the “gene signature score.” To correct for clinical covariates including age and histological grade, we performed multivariable analyses using Cox proportional hazard survival models to obtain the hazard ratio and adjusted *P*‐value.

### 4.13 Cell‐cell interaction analysis

To analyze cell‐cell interactions between different cell types, we used CellPhoneDB^47^ to identify significant ligand‐receptor pairs within early and advanced CRC. The cell type‐specific receptor‐ligand interactions between cell types were identified based on specific expression of a receptor by one cell type and a ligand by another cell type. The interaction score refers to the mean total of all individual ligand‐receptor partner average expression values in the corresponding interacting pairs of cell types. The expression of any complex's output by CellPhoneDB was calculated as the sum of the expression of the component genes.

### 4.14 Statistics and reproducibility

Box plots were generated using the ggplot2 package (version 3.2.0) and default parameters. Hence, the boxes span the interquartile range (IQR; from the 25th to the 75th percentiles), with the centerline corresponding to the median. The upper whisker extends from the hinge to the largest value no further than 1.5× IQR from the hinge (where IQR is the distance between the first and third quartiles). The lower whisker extends from the hinge to the smallest value at most 1.5× IQR of the hinge. Data beyond the end of the whiskers are called “outlying” points and are plotted individually. Each data point is displayed in the box plots.

Comparisons of cell ratios between two groups were performed using unpaired two‐tailed *t*‐tests.

## AUTHOR CONTRIBUTIONS

Xueqing Yao, Zhanglin Lin, Fei Ling, and Shiping Liu jointly supervised the research. Fei Ling, Xueqing Yao, Shiping Liu, Liang Wu, Jianhua Yin, and Wei Wang designed the experiments. Xueqing Yao recruited patients and provided clinical samples from CRC patients. Wei Wang, Yueer Lu, Jiarui Xie, Hang Yu, Shanshan Wang, Chunqing Wang, Yuanhang Zhang, Zhiqiang Jiang, Yilin Huang, Chongyin Han, Zhenggang Zhong, Jialin Hu, Ying Ouyang, Huisheng Liu, Chengzhi Huang, and Mengya Yu prepared the samples under the supervision of Lizhen Huang and Fei Ling. Wei Wang, Yueer Lu, Jiarui Xie, Xiaochan Wei, and Dandan Chen performed all scRNA‐seq data acquisition experiments. Wei Wang, Yu Zhong, Zhenkun Zhuang, Yan Sun, and Jianhua Yin analyzed and interpreted the data. Wei Wang, Yu Zhong, Zhenkun Zhuang, and Yan Sun wrote the manuscript. Zhanglin Lin, Shiping Liu, Wei Wang, Yu Zhong, Zhenkun Zhuang, Chengzhi Huang, and Fei Ling revised the manuscript. All the authors discussed the results and commented on the manuscript.

## CONFLICT OF INTEREST

The authors declare that there is no conflict of interest.

## Supporting information

Figure S1

Table S1

## Data Availability

The data reported in this study are available in the CNGB Nucleotide Sequence Archive (CNSA: https://db.cngb.org/cnsa; accession number: CNP0000916).
