# Supplementary material for: Multiregion single‐cell sequencing reveals the transcriptional landscape of the immune microenvironment of colorectal cancer
Source: Clin Transl Med. 2021 Jan 1;11(1):e253. doi: 10.1002/ctm2.253 (PMC7775989; doi:10.1002/ctm2.253)
Supplement: Supplementary file 1 — Figure S1 [file CTM2-11-e253-s001.pdf]

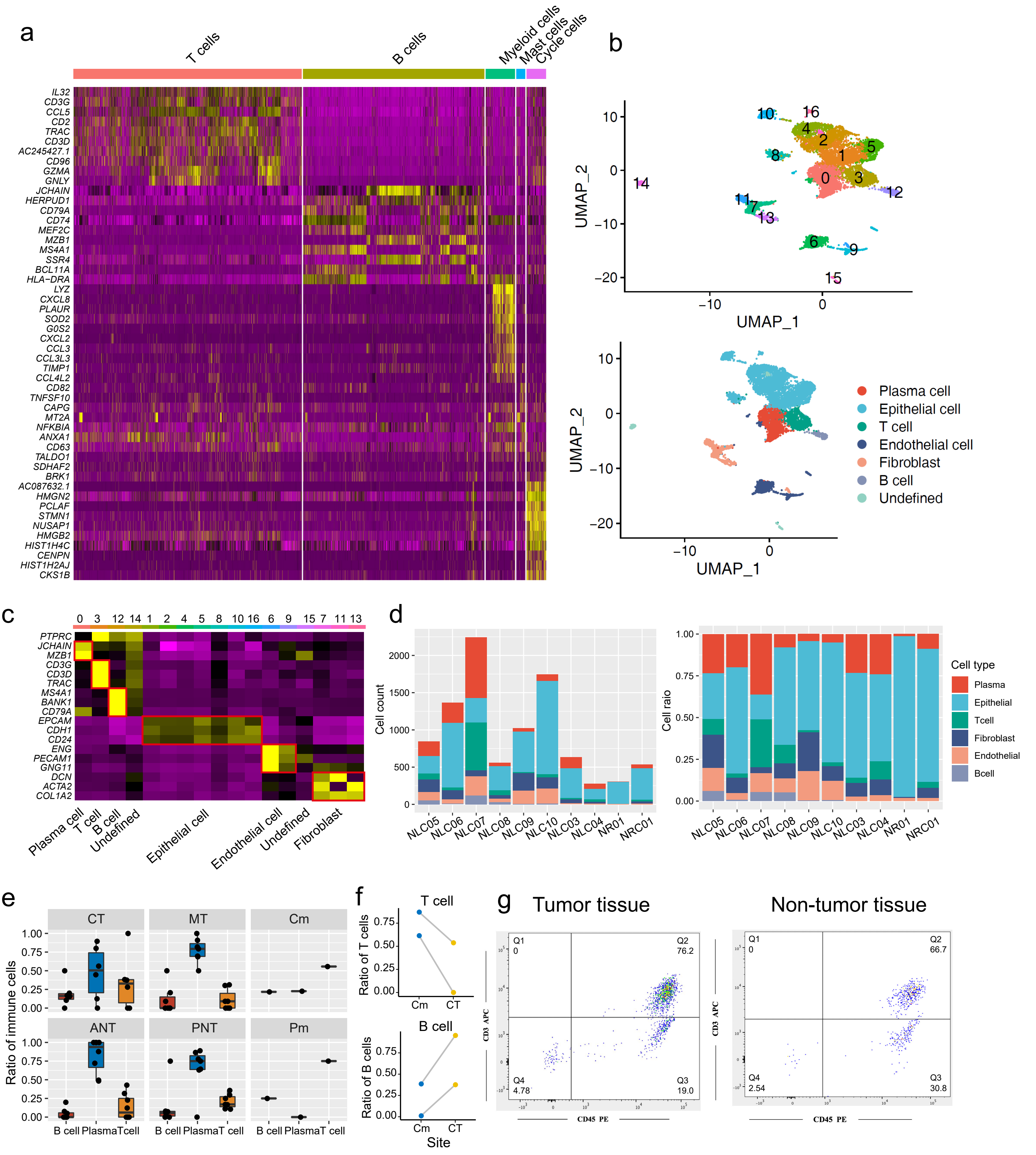

Supplementary Fig. S1 | Diversity of immune cells in CRC. a. Heatmap of the top 15 genes expressed in each cluster as defined in Fig. 1d. **b.** Clustering result of cells from validation cohort. **c.** Heatmap shows the expression of canonical cell markers for cell type identification. **d.** Barplot shows the cell type distribution in each patient. **e.** Boxplot shows the immune cell subtype distribution in different site of samples. **f.** Abundant of T and B cells in primary (CT) and metastatic (Cm) tumor. **g.** Flow cytometry validation of T cells distribution in hepatic metastastic tumor and paried non-tumor tissue from CRC patients.

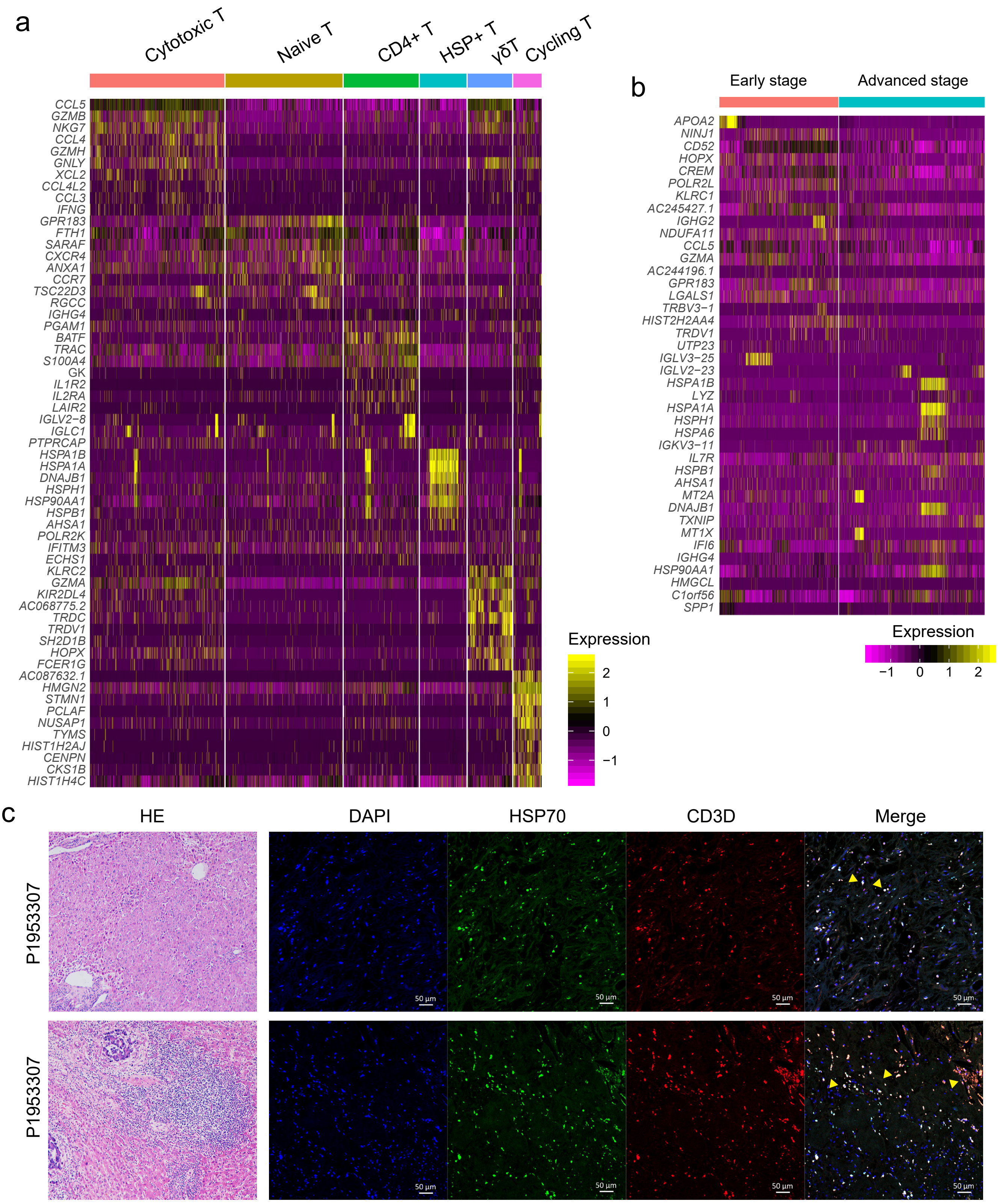

Supplementary Fig. S2 | Heterogeneity of infiltrated T cells in CRC. a. Heatmap of the top 10 discriminative genes expressed in each T cell cluster defined in Fig. 2a. The columns correspond to the cells; the rows correspond to the genes. Cells are grouped by clusters. The color scale is based on a z-score distribution from low (purple) to high (yellow). **b.** Heatmap displaying scaled expression values of genes that best discriminate between early-stage and advanced-stage tumor lesions for all T cells. Best marker genes are sorted by fold change. **c.** Representative HE and IF staining images of hepatic metastases sections from human CRC patients. Left column, HE staining; central to right columns, IF staining. Anti-CD3 and anti-HSP70 were applied to tissues to probe the expression of designated antigens, respectively. Scale bar, 50 μm. Yellow arrows, HSP^{+} T cells.

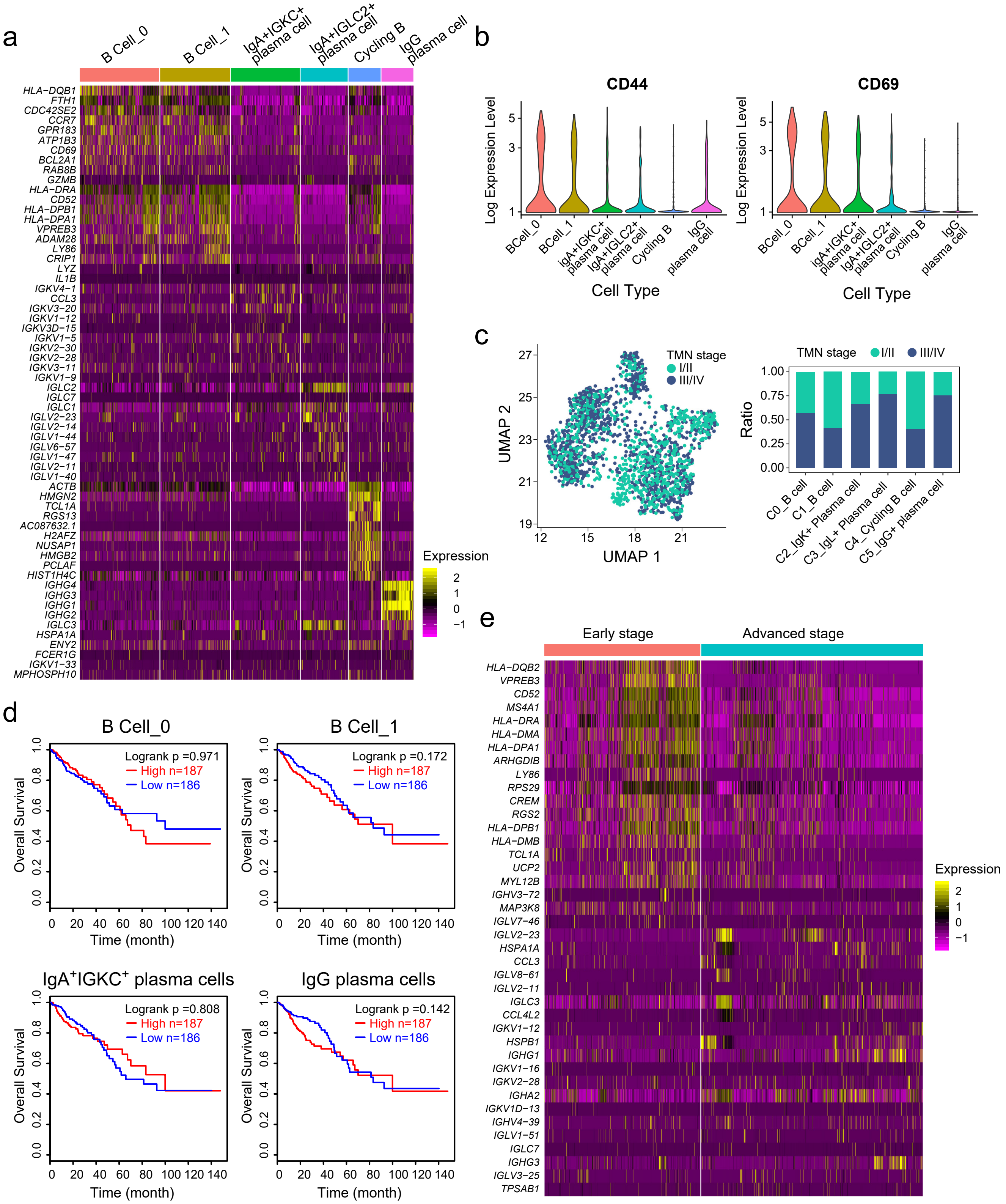

Supplementary Fig. S3 | Heterogeneity of infiltrated B cells in CRC. a. Heatmap of the top 10 discriminative genes expressed in each B cell cluster defined in Fig. 4a. The columns correspond to the cells; the rows correspond to the genes. Cells are grouped by clusters. The color scale is based on a z-score distribution from low (purple) to high (yellow). **b.** Violin plot shows expression of CD44 and CD69 in each C cell subtype. **c.** UMAP plot (top) and batr plot (bottom) show the distribution of of B cells from patients with different TMN stage. **d.** The Kaplan–Meier overall survival curves of TCGA colorectal adenocarcinoma patients grouped by the mean expression of feature of B cell subpopulations. **e.** Heatmap displaying scaled expression values of genes that best discriminate between early-stage and advanced-stage tumor lesions for all B cells. Best marker genes are sorted by fold change.

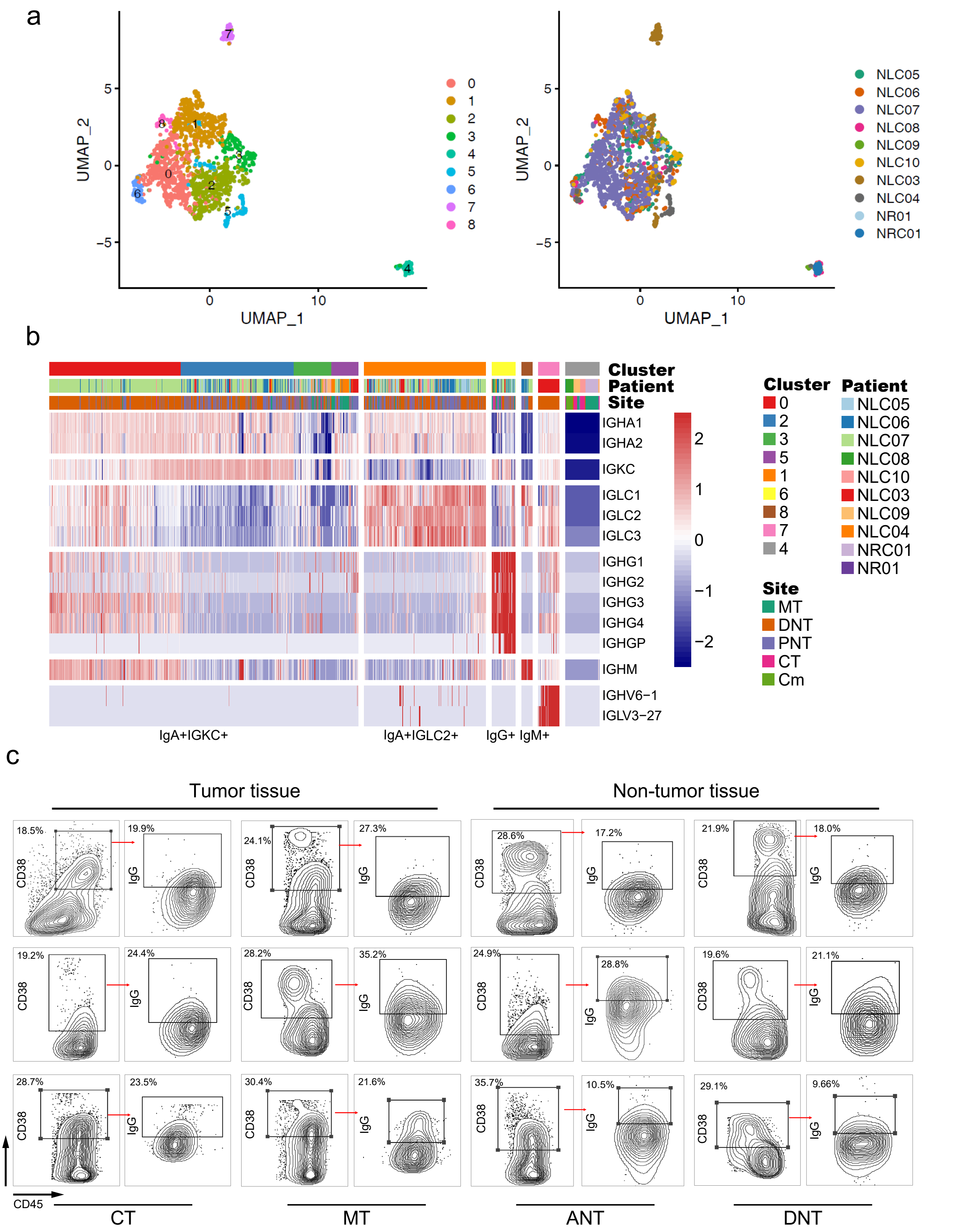

Supplementary Fig. S4 | Plasma cells in CT , MT, ANT and DNT tissue of CRC patients. a. Clustering of plasmas cells from validation cohort. **b.** Immunoglobulin genes expression in each cluster identified in Supplementary Fig. 4a. **c.** The expression of total IgG including the surface and the intracellular IgG in CD38+ B cells in tumor and non-tumor tissues of colorectal cancers.

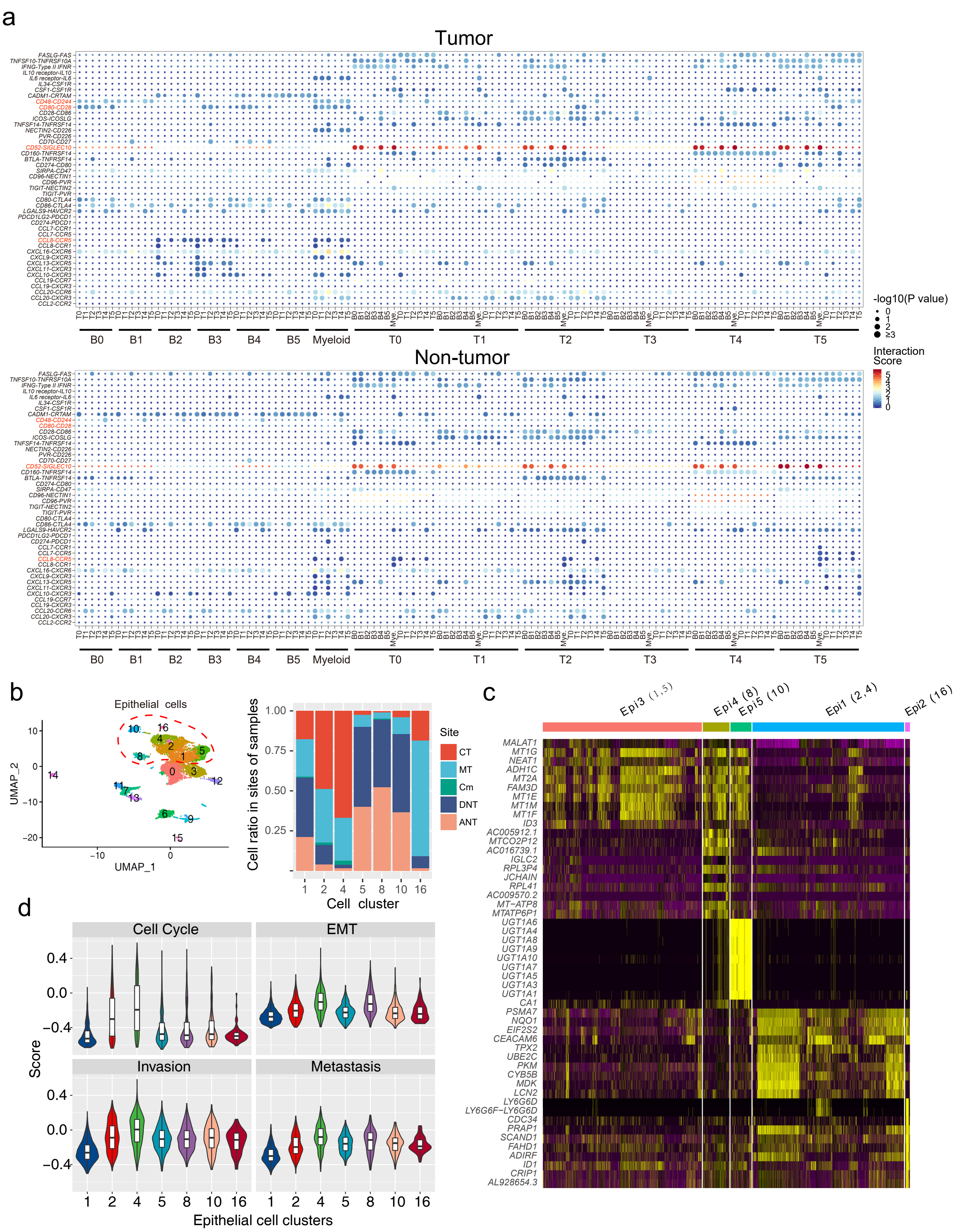

Supplementary Fig. S5 | Immune cell-cell interaction and characters of epithelial. a. Overview of selected cytokine, chemokine and immune check point ligand-receptor interaction pairs in tumor and non-tumor tissue. **b.** Site distribution of epithelial cell cluster. **c.** Heatmap shows top 10 marker genes of each epithelial cell subtype. Number in the parentheses indicats cell cluster as in Supplementary Fig. 5b. **d.** Violin plot shows cell cycle, EMT, invasion and metastasis score of each epithelial cell subtype.

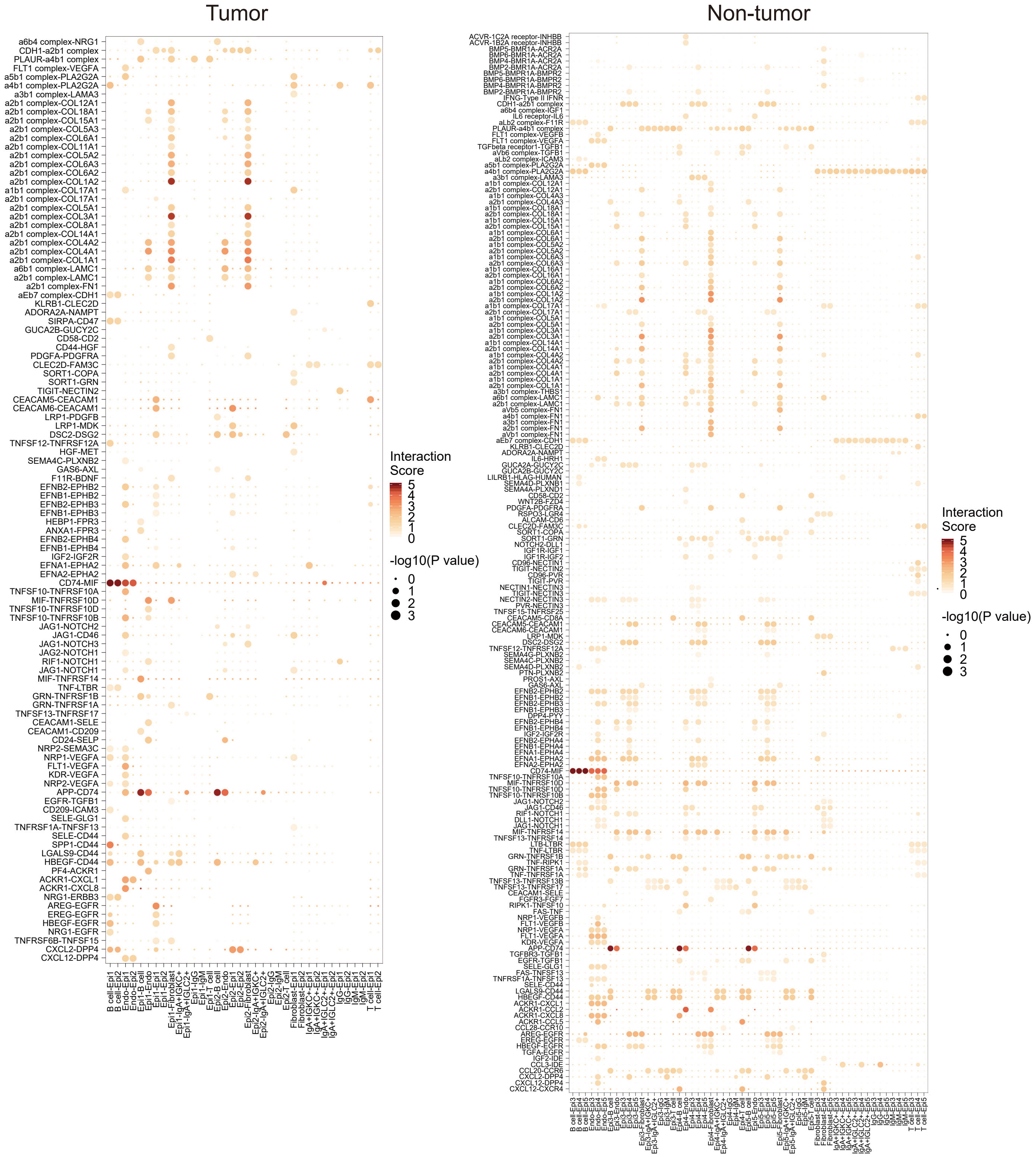

Supplementary Fig. S6 | Overview of Cell-cell interaction between epithelial cells and immune and other stroma cells in tumor and non-tumor tissue from validation cohort.
